# Integrated gene network analysis sheds light on understanding the progression of Osteosarcoma

**DOI:** 10.3389/fmed.2023.1154417

**Published:** 2023-04-04

**Authors:** Hrituraj Dey, Karthick Vasudevan, George Priya Doss C., S. Udhaya Kumar, Achraf El Allali, Alsamman M. Alsamman, Hatem Zayed

**Affiliations:** ^1^Department of Biotechnology, School of Applied Sciences, REVA University, Bangalore, India; ^2^Department of Integrative Biology, School of BioSciences and Technology, Vellore Institute of Technology (VIT), Vellore, India; ^3^African Genome Center, Mohammed VI Polytechnic University, Ben Guerir, Morocco; ^4^Agriculture Genetic Engineering Research Institute (AGERI), Agriculture Research Center (ARC), Giza, Egypt; ^5^International Center for Agricultural Research in the Dry Areas (ICARDA), Giza, Egypt; ^6^Department of Biomedical Sciences College of Health Sciences, QU Health, Qatar University, Doha, Qatar

**Keywords:** Osteosarcoma, gene interaction network, hub genes, TP53, FOXM1 transcription factor

## Abstract

**Introduction:**

Osteosarcoma is a rare disorder among cancer, but the most frequently occurring among sarcomas in children and adolescents. It has been reported to possess the relapsing capability as well as accompanying collateral adverse effects which hinder the development process of an effective treatment plan. Using networks of omics data to identify cancer biomarkers could revolutionize the field in understanding the cancer. Cancer biomarkers and the molecular mechanisms behind it can both be understood by studying the biological networks underpinning the etiology of the disease.

**Methods:**

In our study, we aimed to highlight the hub genes involved in gene-gene interaction network to understand their interaction and how they affect the various biological processes and signaling pathways involved in Osteosarcoma. Gene interaction network provides a comprehensive overview of functional gene analysis by providing insight into how genes cooperatively interact to elicit a response. Because gene interaction networks serve as a nexus to many biological problems, their employment of it to identify the hub genes that can serve as potential biomarkers remain widely unexplored. A dynamic framework provides a clear understanding of biological complexity and a pathway from the gene level to interaction networks.

**Results:**

Our study revealed various hub genes viz. TP53, CCND1, CDK4, STAT3, and VEGFA by analyzing various topological parameters of the network, such as highest number of interactions, average shortest path length, high cluster density, etc. Their involvement in key signaling pathways, such as the FOXM1 transcription factor network, FAK-mediated signaling events, and the ATM pathway, makes them significant candidates for studying the disease. The study also highlighted significant enrichment in GO terms (Biological Processes, Molecular Function, and Cellular Processes), such as cell cycle signal transduction, cell communication, kinase binding, transcription factor activity, nucleoplasm, PML body, nuclear body, etc.

**Conclusion:**

To develop better therapeutics, a specific approach toward the disease targeting the hub genes involved in various signaling pathways must have opted to unravel the complexity of the disease. Our study has highlighted the candidate hub genes viz. TP53, CCND1 CDK4, STAT3, VEGFA. Their involvement in the major signaling pathways of Osteosarcoma makes them potential candidates to be targeted for drug development. The highly enriched signaling pathways include FOXM1 transcription pathway, ATM signal-ling pathway, FAK mediated signaling events, Arf6 signaling events, mTOR signaling pathway, and Integrin family cell surface interactions. Targeting the hub genes and their associated functional partners which we have reported in our studies may be efficacious in developing novel therapeutic targets.

## 1. Introduction

The prominence of Osteosarcoma dates back to the early nineteenth century when the French surgeon Alexis Boyer first coined the term and William Enneking described the disease. A recent study by the American Cancer Society found that 186.6 per 100,000 children and adolescents were diagnosed with cancer each year from birth to age 19 (1). Osteosarcoma is the most common type of bone cancer, originating in the mesenchyme tissue. The tumor usually develops around the pelvis or long bone and then metastasizes to neighboring tissue (2). The most prevalent locations in femur (42%, of which 75% is in the distal femur), the tibia (19%, of which 80% is in the proximal tibia), and the humerus (10%). The jaw or skull (8%) and the pelvis (8%) are additional potential sites. In the ribs, Osteosarcomas only comprise 1.25 percent of cases (3), (4).

Although it is seen in both young and adults, it has been observed that the tumor spreads rapidly when the bone undergoes the stages of its growth. It has a bimodal age distribution with an adolescence and elderly peak in incidence. The incidence often peaks between the ages of 10 and 14 years, after which it starts to subside. Adults over 65 see the second peak in Osteosarcoma incidence, more likely to be a second malignancy commonly linked to Paget disease (5). The genomic landscape of Osteosarcoma based on various sequencing methods revealed that alterations in the sequence are due to somatic point mutations such as single base substitutions, insertions, and deletions. Other structural variants such as rearrangements and somatic copy number alterations leading to copy number decrease may downregulate a tumor suppressor gene driver and copy number increase may trigger an oncogene driver (6, 7). Numerous familial syndromes are associated with Osteosarcoma. Li-Fraumeni syndrome is one such condition with a high prevalence of Osteosarcoma. This condition is characterized by various malignancies, including leukemia, breast, sarcoma, adrenocortical, and brain tumors (8). It is an autosomal dominant disorder where the p53 tumor suppressor gene is rendered inactive, which helps advance the cell cycle in the presence of DNA damage.

Additionally, it has been demonstrated that alteration in additional p53 pathway genes, such as *MDM2, p14ART, and CDK4*, may increase a person's risk of acquiring Osteosarcoma (9). DNA helicase anomalies have also been reported in Osteosarcoma. In the autosomal recessive disorder, Rothman-Thomas syndrome, which is associated with skin changes, alopecia, and Osteosarcoma, gene *RECQL4* coding for DNA helicase is found to be defective. Similar DNA helicase aberrations are found in Werner syndrome where the *WRN or RECQL4* gene is defective causing melanoma, Osteosarcoma, etc. (10).

During the mid-1970s, chemotherapy was shown to be adequate for treating Osteosarcoma. Osteosarcoma is typically treated with neoadjuvant chemotherapy that includes cisplatin, doxorubicin, ifosfamide, and high-dose methotrexate given along with leucovorin. This is followed by surgical resection and adjuvant chemotherapy (11). Although the current treatment regime has proven to be partially effective, it is associated with short- and long-term concomitant side effects such as accumulating toxic compounds in other organs such as the liver, kidney, heart, etc., leading to other detrimental effects on the body. For instance, higher dosage rates were linked to an increased risk of nephrotoxicity and gonadal dysfunction brought on by cisplatin. The dosage intensity and the total dose of doxorubicin were associated with an increased risk of cardiac toxicity (12). Thus, the hub genes involved in the various enriched biological processes and signaling pathways must be identified to develop better treatment strategies. These hub genes are essential because they play a role in regulating the molecular mechanism. Our study aimed to highlight the hub genes involved in the gene-gene interaction network to understand their interaction and how they affect the various biological processes and signaling pathways involved in Osteosarcoma. Using networks of omics data to identify cancer biomarkers could revolutionize the field. Cancer biomarkers and the molecular mechanisms behind it can both be understood by studying the biological networks underpinning the disease (13). Several network-based analysis tools were used for biomarker identification in recent years. For instance, a gene co-expression network (GCN) was developed to effectively identify biomarkers in glioma. It was also utilized to assess a gene module relevant to lung cancer, predictive biomarkers for estrogen receptor-positive breast cancer treated with tamoxifen, and biomarkers for anticipating the chemotherapy response in breast cancer (14–16). In our earlier studies, we have used advanced computational tools to decipher and predict the pathogenicity of the various diseases (17, 18). Gene interaction network provides a broad view of functional gene analysis by giving an insight into how genes cooperatively interact to elicit a response. A dynamic framework offers a clear understanding of biological complexity and a pathway from the gene level to interaction networks. The term “interaction” refers to the relationship between genes that can affect other genes' operations. Because gene interaction networks serve as a nexus to many biological problems, their employment of it to identify the hub genes that can serve as potential biomarkers remain widely unexplored. Gene interaction network assists in identifying novel candidate genes, based on the idea that the neighboring genes located near the disease-causing gene in a network are more likely to cause a similar disease (19).

## 2. Materials and methods

### 2.1. Cancer genetics web server

The Cancer genetics web server is an online resource portal, which provides information on various cancers, particularly for researchers and health professionals exploring this field. Sever is available at www.cancer-genetics.org/. Using PubMed, the data were obtained by utilizing information from numerous data sources and literary reviews. It offers comprehensive links to credible information about genes, their associated proteins, and genetic alterations linked to cancer and related disorders. The site includes a directory of genes identified as the oncogenes and the tumor suppressor genes. Every gene page includes accessible links to major genetic databases and abstracts, references, external searches, and summary information wherever possible.

### 2.2. STRING database

STRING (Search tool for retrieval of the interacting gene) (https://string-db.org/) is an online, publicly accessible database harboring information on protein-protein interaction. The interactions include direct as well as indirect connections. It provides a versatile way for analyzing and visualizing the data, such as setting confidence scores that reflect the level of interaction, no of interactors, network type, display mode, etc. In order to categorize the interactions, String uses the confidence scores: highest (above 0.90), high (0.7–0.89), medium (0.4–0.69), and low (0.15–0.39). The STRING database accepts the input in various forms, such as protein by name, protein by sequence, multiple proteins, protein families (COG), etc. The outcome of the network can be saved in a variety of formats such as bitmap image, vector graphic, TSV format, tab-delimited file, etc. (20).

### 2.3. Cytoscape

Gene interaction networks can be visualized and analyzed using Cytoscape (https://cytoscape.org/). It provides a user-friendly interface that allows the user to seamlessly operate the software. It supports various plugins, which serve various purposes such as clustering of genes, enrichment analysis, annotation, determining topological properties of a network, etc. Output from STRING was used as an input for the Cytoscape.

#### 2.3.1. Network analyzer

It is a plugin in Cytoscape that calculates topological parameters in a network. Numerous parameters can be computed, such as degree, number of nodes, edges, average no. of neighbors, clustering coefficient, average shortest path length, closeness centrality, and betweenness centrality. The degree and average shortest length are the essential parameters while analyzing the network since the degree represents the direct interactors of the desired gene, whereas the average shortest path is the distance between two nodes. Closeness centrality measures how fast information travels from one node to another node in a network, whereas betweenness centrality represents the degree of influence a node exerts upon other interactions of a node (21). The results generated can be exported as a CSV file or directly analyzed in the software.

#### 2.3.2. MCODE

MCODE is a plugin used to identify clusters in a network. Clusters are highly interrelated regions that are grouped in a network. The MCODE method is based on analyzing densely interconnected regions where nodes have more interconnected nodes, detecting potential clusters, and evaluating the number of interconnected nodes (node scoring). Genes are clustered by MCODE based on their connectivity, in which the same cluster contains more interconnected genes with the optimal neighborhood density. Genes that are associated with MCODE scores are clustered together. (22).

### 2.4. FunRich

FunRich (http://www.funrich.org/) is a tool used for functional enrichment and network analysis. It can be utilized to conduct functional enrichment analysis on background databases incorporating diverse genomic and proteomic resources. The outcomes of the enrichment studies may be depicted using a wide range of graphical layouts, such as column graphs, bar graphs, pie charts, Venn diagrams, heat maps, and doughnut charts. Users can download information from the UniProt and standard human-specific FunRich databases. Additionally, users can create their custom datasets and carry out enrichment analyses regardless of the organism (23).

## 3. Results

### 3.1. Data collection

**Figure 1:**
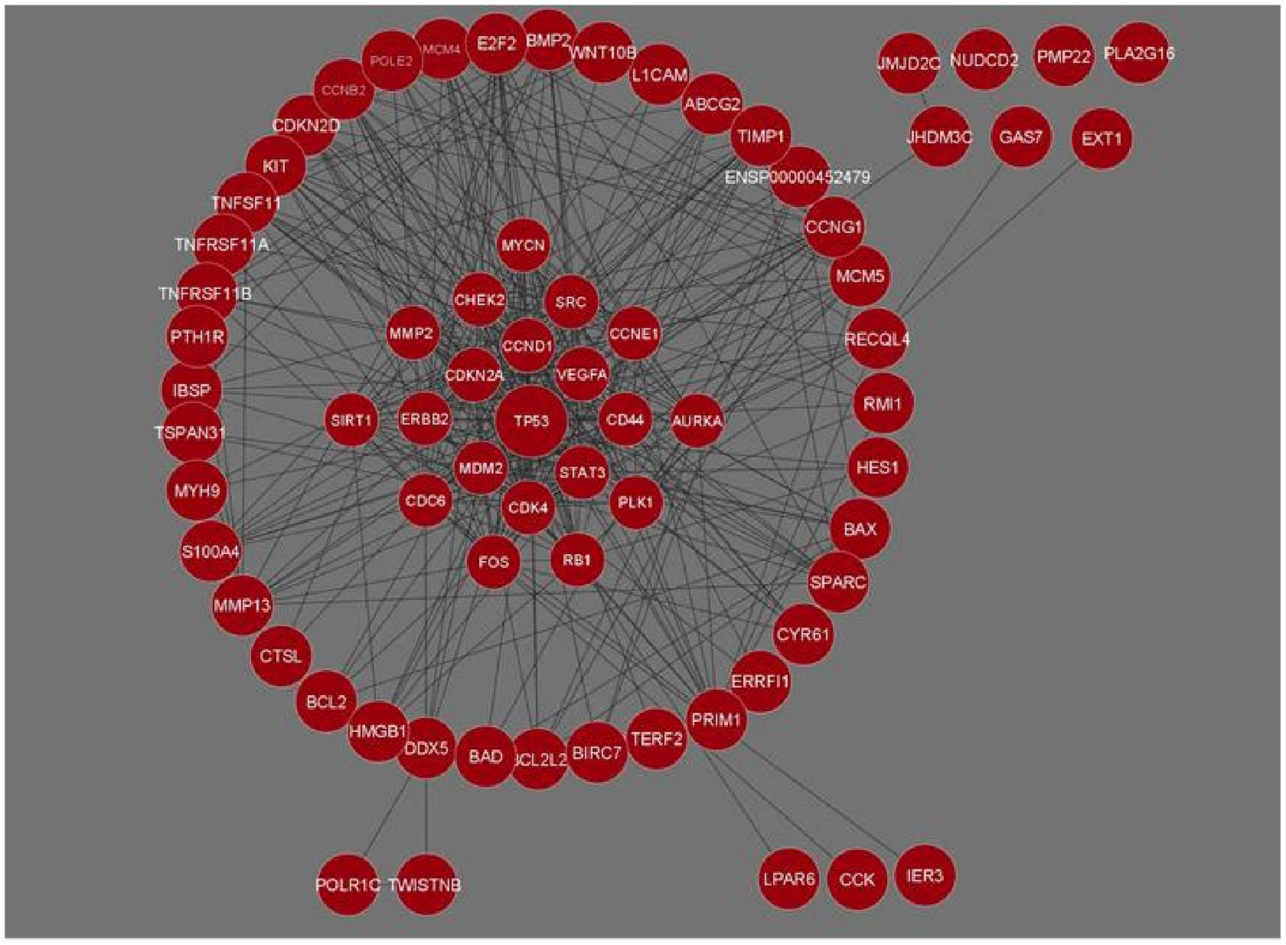
Gene interaction network of Osteosarcoma comprising 71 genes and 426 interactions built in Cytoscape. Genes with the maximum number of network interactions are positioned in the center (ENSP00000452479 is the Ensembl protein ID for sequence BCL2L2-PABPN1).

The genes for Osteosarcoma responsible for its growth and development were curated from Cancer genetics web database. The sites host information on genes for 76 different cancers and associated conditions. The information on genes related to Osteosarcoma was searched based on the keywords. We were able to gather 58 genes and their related information. This data was used for a STRING interaction network. The interaction network was maximized, with a medium confidence score (0.4) which gave an interaction for 71 genes and their functional partners. Gene networks were constructed and further analyzed based on STRING interaction data (Figure 1).

### 3.2. Network analysis

The network analysis of 71 genes was carried out using NetworkAnalyzer. To study the gene interaction network, it analyzed different topological parameters such as degree, no of nodes and edges, characteristic path length, clustering coefficient, closeness centrality, and betweenness centrality. The top genes with the highest degree values are *TP53, CCND1, CDK4*, and *STAT3* with no interactors 45, 33, 28, and 27, respectively. Table 1 lists the 20 genes along with their various analyzed parameters. The network analysis revealed the no. of nodes to be 71 and no. of edges to be 426, while the clustering coefficient of the entire network was 0.583.

**Table 1:** The list of the top 20 genes in Osteosarcoma analyzed by NetworkAnalyzer.

**Genes**	**Degree**	**Avg. shortest path length**	**Closeness centrality**	**Betweenness centrality**
*TP53*	45	1.343283582	0.744444	0.278506532
*CCND1*	33	1.582089552	0.632075	0.076460459
*CDK4*	28	1.626865672	0.6146788	0.047913393
*STAT3*	27	1.71641791	0.587798	0.033714855
*VEGFA*	27	1.701492537	0.582606	0.033879065
*CDKN2A*	27	1.641791045	0.609009	0.037095448
*MDM2*	26	1.701492537	0.587718	0.041774175
*SRC*	25	1.74626865	0.572643	0.053286042
*CHEK2*	24	1.746268657	0.572643	0.023052737
*ERBB2*	23	1.776119403	0.567713	0.036390641
*RB1*	23	1.76119403	0.563020	0.080214837
*FOS*	23	1.76119403	0.567794	0.051156614
*CD44*	22	1.805970149	0.553719	0.014110304
*CCNE1*	21	1.835820896	0.544715	0.007678749
*PLK1*	20	1.880597015	0.544715	0.025338494
*MMP2*	20	1.835820896	0.531746	0.009431072
*CDC6*	20	1.835820896	0.544715	0.075482361
*MYCN*	19	1.925373134	0.51937	0.012188444
*AURKA*	19	1.835820896	0.544715	0.004759471
*SIRT1*	18	1.776119403	0.563028	0.020802171

### 3.3. Clustering analysis

**Figure 2:**
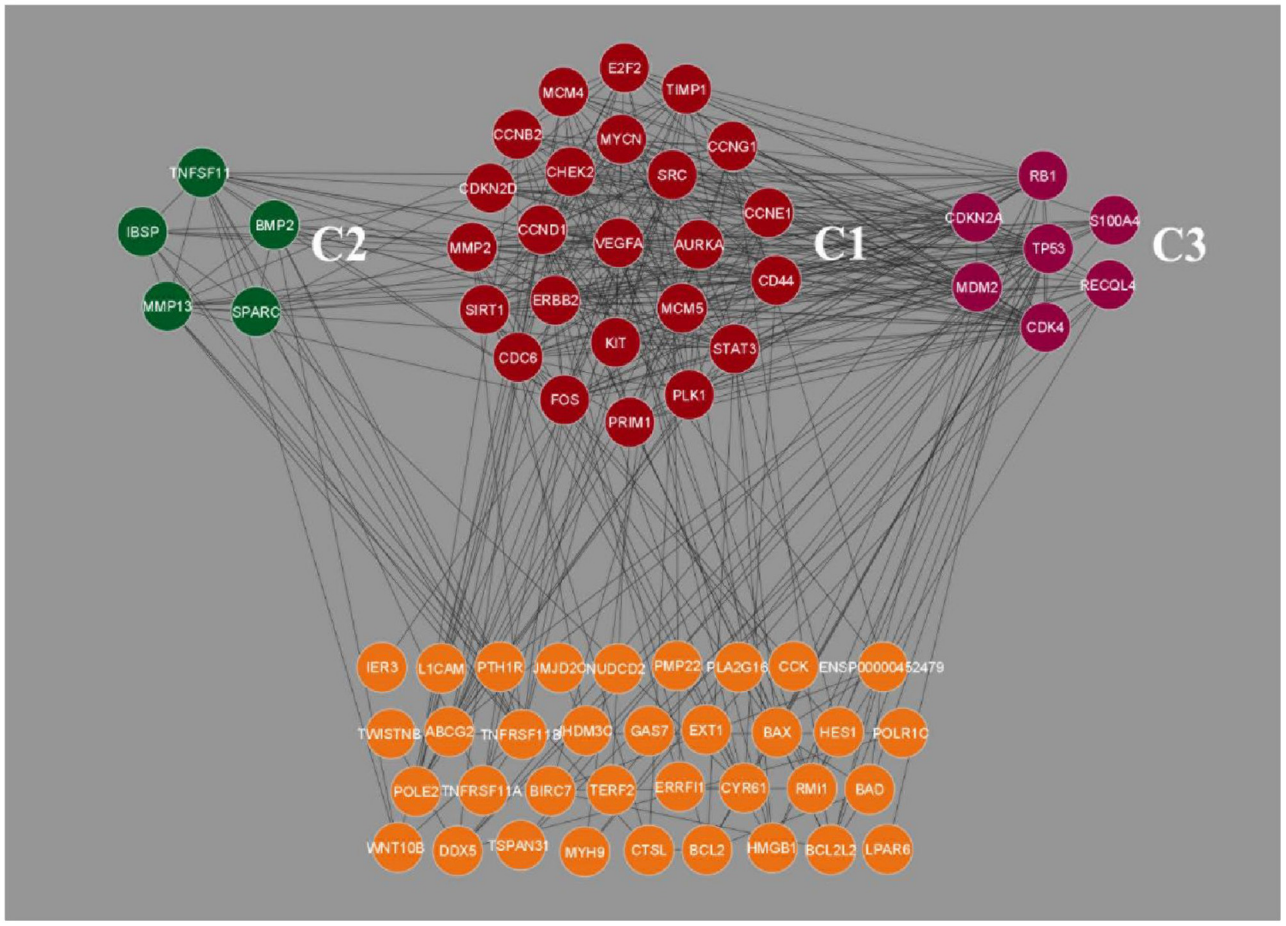
Clustering analysis of Osteosarcoma gene interaction network using MCODE. The genes were grouped into three clusters, *viz*. C1, C2 and C3. Cluster C1 showed the highest level of clustering, followed by C2 and C3. The unclustered genes are located beneath, highlighted in orange color.

Clustering analysis of the gene interaction network was done using MCODE, resulting in the genes in a cluster of 3. *viz*. C1, C2, and C3 (Figure 2). The clustering of genes allowed us to understand the highly interconnected regions. Clustering of MCODE is influenced by both directed interactions and interactions between the associated interactors. Out of 71 genes in the network, 36 are identified as part of the cluster. Among the three, cluster C1 had the most inter-connected regions constituting 24 nodes and 137 edges with an MCODE score of 11.913, followed by C2 with five nodes and ten edges with a score of 5.0, and C3 with 7 nodes and 14 edges with a score of 4.667 (Table 2).

**Table 2:** List of Osteosarcoma related genes and their associated signaling pathways.

**Signaling pathways**	**Genes**
FOXM1 transcription factor network	*CCND1, CDK4, CDKN2A, CHEK2, RB1, FOS, CCNE1, PLK1, MMP2*, and *CCNB2*
FAK-mediated signaling events	*TP53, CCND1, CDK4, STAT3, VEGFA, CDKN2A, MDM2, SRC, CHEK2, ERBB2, RB1, FOS, CCNE1, PLK1, MMP2, SIRT1, E2F2, CCNG*, and *TNFSF11*
ATM pathway	*TP53, CDKN2A, MDM2, CHEK2, RB1, CCNE1, PLK1, MMP2, CDC6, SIRT1, E2F2*, and *CCNG1*
Arf6 signaling events	*TP53, CCND1, CDK4, STAT3, VEGFA, CDKN2A, MDM2, SRC, CHEK2, ERBB2, RB1, FOS, CCNE1, PLK1, MMP2, SIRT1, E2F2, CCNG1*, and *TNFSF11*
Class I PI3K signaling events	TP53, CCND1, CDK4, STAT3, VEGFA, CDKN2A, MDM2, SRC, CHEK2, ERBB2, RB1, FOS, CCNE1, PLK1, MMP2, SIRT1, E2F2, CCNG1, and TNFSF11
mTOR signaling pathway	*TP53, CCND1, CDK4, STAT3, VEGFA, CDKN2A, MDM2, SRC, CHEK2, ERBB2, RB1, FOS, CCNE1, PLK1, MMP2, SIRT1, E2F2, CCNG1*, and *TNFSF11*
EGF receptor(ErbB1) signaling pathway	*TP53, CCND1, MDM2, SRC, CHEK2, ERBB2, RB1, FOS, CDK4, STAT3, VEGFA, CDKN2A, CCNE1, PLK1, MMP2, SIRT1, E2F2, CCNG1*, and *TNFSF11*
Integrin family cell surface interactions	*TP53, CCND1, CDK4, STAT3, VEGFA, CDKN2A, MDM2, SRC, CHEK2, ERBB2, RB1, FOS, CCNE1, PLK1, MMP2, SIRT1, E2F2, CCNG1*, and *TNFSF11*

### 3.4. Functional enrichment analysis

**Figure 3:**
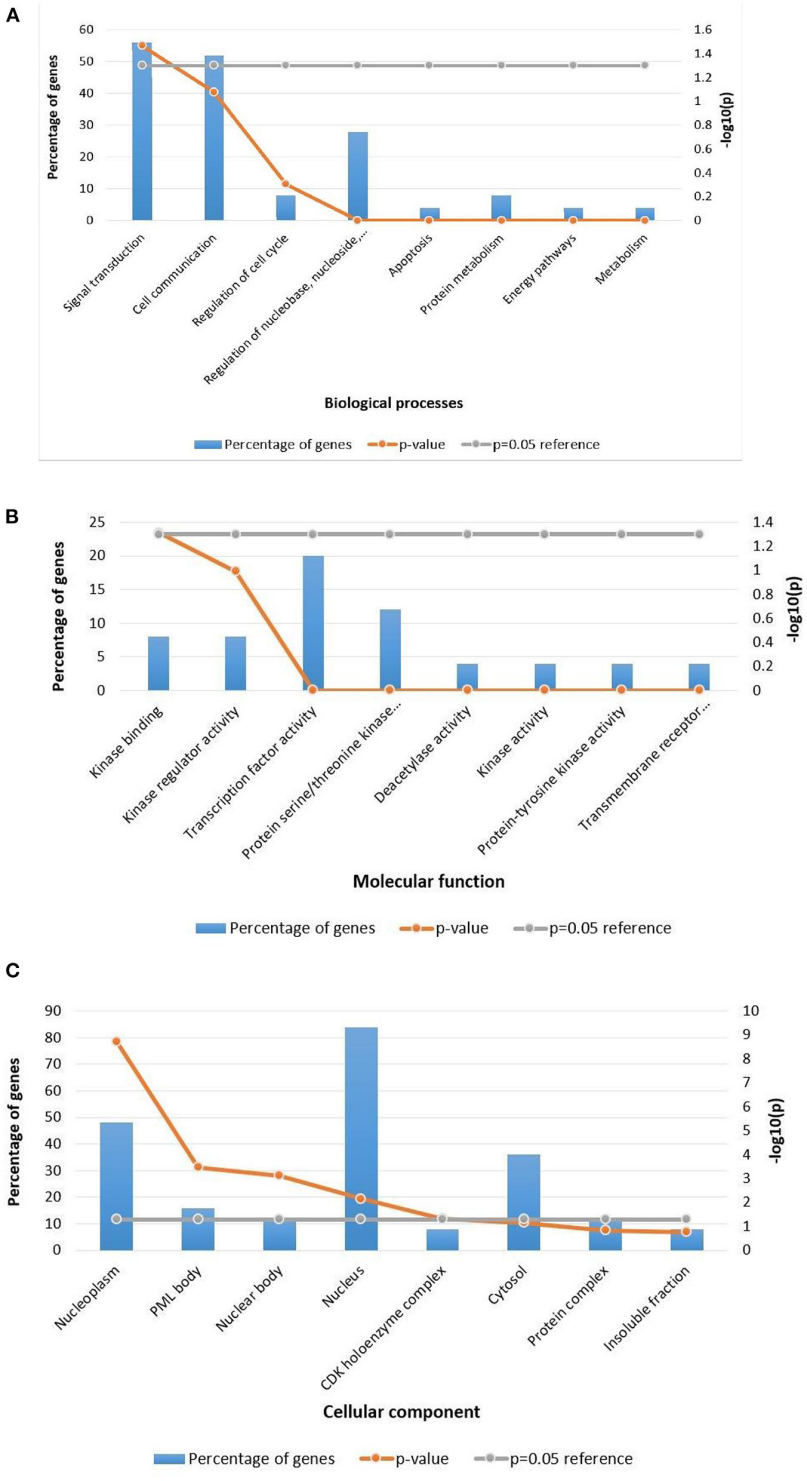
Functional enrichment analysis of Osteosarcoma gene. Significantly enriched **(A)** Biological processes, **(B)** Molecular function, **(C)** Cellular component. The *p*-value is taken as 0.05 for reference.

**Figure 4:**
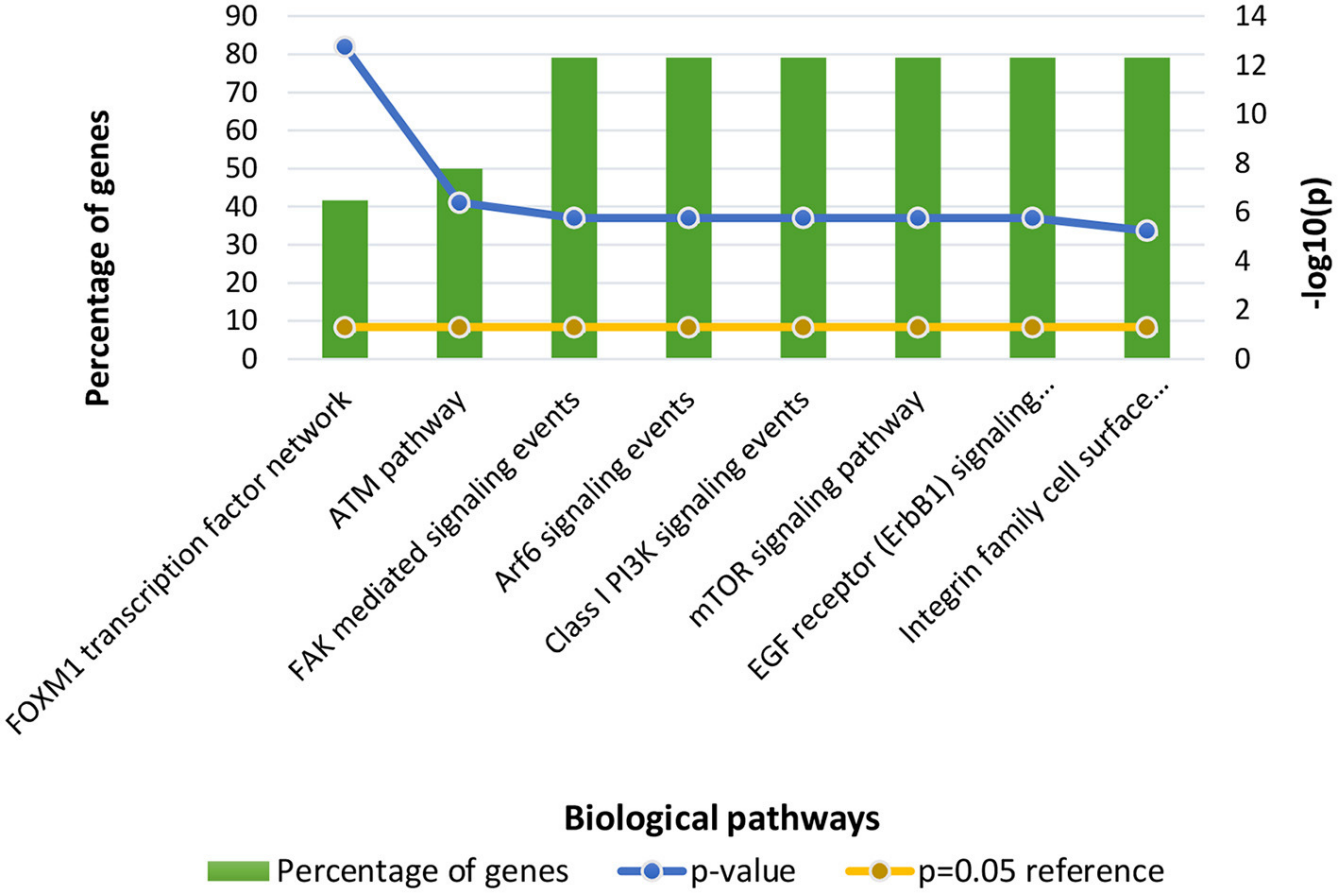
Functional enrichment of Osteosarcoma genes involved in significant biological pathways. The *p*-value is considered 0.05 for reference.

Following clustering analysis, functional enrichment analysis was performed using the STRING database and FunRich tool, clarifying genes' contribution to various processes and pathways. The Bonferroni correction method obtained Gene ontology terms with a *p*-value ≤ 0.05. Using the Bonferroni correction, multiple comparisons are compensated by dividing the significance level by the number of comparisons. A significance level indicates the likelihood that a given test will detect an incorrect difference in the sample that does not exist in the population (false positive). Commonly, significance levels of 0.05 are considered significant. The genes observed in Osteosarcoma revealed various contributions in Gene Ontology terms such as Biological Processes (BP), Molecular Function (MF), and Cellular Compartment (CC). The significantly enriched terms in BP included regulation of cell cycle signal transduction, cell communication, regulation of nucleobase, nucleoside, nucleotide, and nucleic acid metabolism (Supplementary File 1), MF included kinase binding, kinase regulator activity, transcription factor activity (Supplementary File 2) and CC included nucleoplasm, PML body, nuclear body, nucleus, and cytosol (Supplementary File 3; Figure 3). The enriched signaling pathways is of utmost importance while studying the progression of cancer. Cancer involves various signal transmission pathways, which promote its progression. The signaling pathways involved in tumor progression of Osteosarcoma are the FOXM1 transcription pathway, ATM signaling pathway, FAK mediated signaling events, Arf6 signaling events, Class 1 PI3K signaling events, mTOR signaling pathway, and Integrin family cell surface interactions (Supplementary File 4; Figure 4). The genes involved in various signaling pathways of Osteosarcoma are mentioned in Table 3.

**Table 3:** Clustering analysis of Osteosarcoma gene interaction network.

**Cluster**	**MCODE score**	**No. of nodes**	**No. of edges**	**Node IDs**
C1	11.913	24	137	*VEGFA, MYCN, SRC, AURKA, MCM5, KIT, ERBB2, CCND1, CHEK2, E2F2, TIMP1, CCNG1, CCNE1, CD44, STAT3, PLK1, PRIM1, FOS, CDC6, SIRT1, MMP2, CDKN2D, CCNB2*, and *MCM4*
C2	5.00	5	10	*TNFSF11, BMP2, SPARC, MMP13*, and *IBSP*
C3	4.677	7	14	*TP53, RB1, S100A4, RECQL4, CDK4, MDM2*, and *CDKN2A*

## 4. Discussion

A cancer cell will essentially have six hallmark capabilities to be recognized as a cancer cell. The six core hallmarks outlined by Hananah and Weinberg include self-sufficiency in growth signals, insensitivity to antigrowth signals, evasion of programmed cell death (apoptosis), limitless replicative potential, sustained angiogenesis, and tissue invasion and metastasis, along with the emerging hallmarks of cancer which includes deregulating cellular energetics and avoiding immune destruction (24, 25). Attaining each capability will likely involve inactivating or eluding a specific control mechanism. We have utilized a gene interaction network in our study to understand the development and progression of the tumor cells in Osteosarcoma. This helped us decipher a group of highly interactive genes responsible for the pathogenesis and spread of the disease.

During analysis, MF observed were kinase binding, kinase regulator activity, and transcription factor activity. Prior studies on Osteosarcoma have highlighted that protein tyrosine kinases are essential signaling molecules involved in the signaling pathways that regulate cellular differentiation and proliferation (26). The enriched BPs of Osteosarcoma included signal transduction, cell communication, regulation of cell cycle, regulation of nucleobase, nucleoside, nucleotide and nucleic acid metabolism, apoptosis, protein metabolism, energy pathways, metabolism along with cell cycle checkpoint signaling, DNA damage checkpoint signaling, and response to hypoxia. Earlier studies have shown that impairment in signal transduction, cell communication, and cell cycle checkpoint signaling has significantly promoted Osteosarcoma (27). Signal transduction is a sequential event where an extracellular signal is transduced by the cell to create a response, which is necessary for the normal growth and development of the cell. Since genetic alterations drive cancer, these alterations create a wide range of aberrant signaling networks that drives the expansion of the tumor. These signaling pathways control tumor growth, development, and fate (28). The signal transduction pathway involved 14 genes namely *CCND1, CDK4, VEGFA, CDKN2A, SRC, CHEK2, ERBB2, CD44, CCNE1, PLK1, CDC6, AURKA, CCNB2*, and *TNFSF11*. It has been reported that patients suffering from Osteosarcoma cells develop resistance toward the kinase inhibitor drug, Sorafenib due to the mTOR signaling pathway. The mammalian target of rapamycin (mTOR) facilitates all cell proliferation, apoptosis, and autophagy. There is evidence showing that the mTOR signaling pathway plays a significant role in a number of diseases, including osteosarcoma. (29). The mTOR is structurally made up of a dimer complex called the mammalian target of rapamycin complex 1 (mTORC1) and the mammalian target of rapamycin complex 2 (mTORC2) (30). mTORC1 has been mostly seen in controlling cell growth and metabolism, while mTORC2 primarily governs cell proliferation and survival (31). Numerous signaling pathways in the body, such as phosphoinositide-3-kinase (PI3K)/AKT, tuberous sclerosis complex subunit 1 (TSC1)/tuberous sclerosis complex subunit 2 (TSC2)/Rheb, LKBL/adenosine 5′monophosphate-activated protein kinase (AMPK), VAM6/Rag GTPases, and others, are regulated by mTOR (32). Under normal circumstances, mTOR plays a significant role in regulating cell growth and division. However, it is hyper-activated in tumor cells sending aberrant signals that help tumor cells grow and proliferate, thus promoting malignancy (33). mTOR pathway incessantly activates the AKT signaling pathway among the other pathways (34). Our study revealed 19 genes involved in the mTOR signaling pathway of Osteosarcoma viz. *TP53, CCND1, CDK4, STAT3, VEGFA, CDKN2A, MDM2, SRC, CHEK2, ERBB2, RB1, FOS, CCNE1, PLK1, MMP2, SIRT1, E2F2, CCNG1*, and *TNFSF11*. The involvement of mutated genes *TP53* and *VEGFA* is closely associated with all types of cancer. *TP53* controls cell growth and proliferation by acting as a tumor suppressor gene. The alteration in the sequence of TP53 leads to tumor development. *VEGFA* promotes the mTOR signaling in Osteosarcoma by promoting angiogenesis in the tumor (35). It has been studied that there is significant upregulation in mTORC1 during tumor growth and development, and mTORC1 is comparatively more sensitive to rapamycin than mTORC2. Thus, rapamycin acts as an inhibitor of mTOR (36). Different approaches can be sought, such as down-regulating the mTOR complexes to control cell proliferation. Because of its close linkage with Osteosarcoma, mTOR pathways, and the associated genes can serve as a therapeutic target for the disease. Cell communication was also seen to be significantly enriched in the Biological Processes. Communication between the neighboring cells is crucial for normal cellular activities. Numerous studies have demonstrated that a complex intercellular communication system, whether through direct cell-to-cell contact or traditional paracrine/endocrine signaling, plays a crucial role in the growth and expansion of tumors (37). The most basic signal transmission to the proximal or the distant cells is the release of soluble substances into the extracellular space, such as cytokines, chemokines, and growth factors. Along with it, another cell interaction involves adhesion molecules and gap junction (38). Recent studies have also demonstrated that healthy mitochondria and other organelles may be donated by non-cancer cells through tunnel nanotubes to keep cancer cells alive, but it has also been revealed that horizontal mitochondrial transfer from cancer cells to neighboring cells is equally possible (39).

The Integrin family of proteins binds extracellular matrix ligands and cell-surface ligands to act as cell adhesion receptors during cell communication. Our study of Osteosarcoma significantly enriches the integrin family of cell surface interactions. Integrins connect with the extracellular matrix (ECM) via the extracellular domain, supplying anchoring (40). Integrins are also responsible for transmitting chemical signals into the cells, where the signals develop in the ECM after ligation and involve receptor clustering and binding of a particular ligand (41). As a response to this clustering and the presence of GTPase Rho A, cytoskeletal proteins like focal adhesion kinase (FAK) are formed. The Ras protein, which plays a crucial role in cell signaling and gene expression, is phosphorylated by FAK to activate the mitogen-activated protein (MAP) kinase pathway (42). FAK plays a role in co-localizing with integrin receptors in adherent cell types at cell-substratum contact points known as focal adhesions (43). FAK stimulates cell motility, survival, and proliferation through kinase-dependent and -independent processes during the development of various malignancies (44). Studies have reported that FAK signaling is located at the junction of other signaling pathways promoting metastasis (45). According to various reports, FAK signaling is linked to the maintenance of cancer stem cells (46). It has been highlighted that the tumor cells of Osteosarcoma interact with their microenvironment, where β4 integrin plays a significant role in metastasis and the invasive nature of cancer (47). Growth factors and integrin ligands work synergistically to regulate the differentiation of osteogenic cells from stem cells (48). Growth factors called Bone Morphogenic Proteins (BMPs) substantially impact the development and remodeling of postnatal skeletal tissue, among other things (49). There are 14 known BMPs, collectively constituting a subfamily with Growth Differentiation Factors (GDFs). Among the 14 known BMPs, BMP-2, BMP-4, BMP-6, BMP-7, and BMP-9 are especially important as they have been found to induce complete bone morphogenesis (50). It has been reported that the inhibition of β4 integrin has gradually mitigated and inhibited metastasis in patients with Osteosarcoma (51). Thus, analyzing the network targeting genes involved in the integrin family of cell surface interactions can help develop therapeutic targets for the disease.

**Figure 5:**
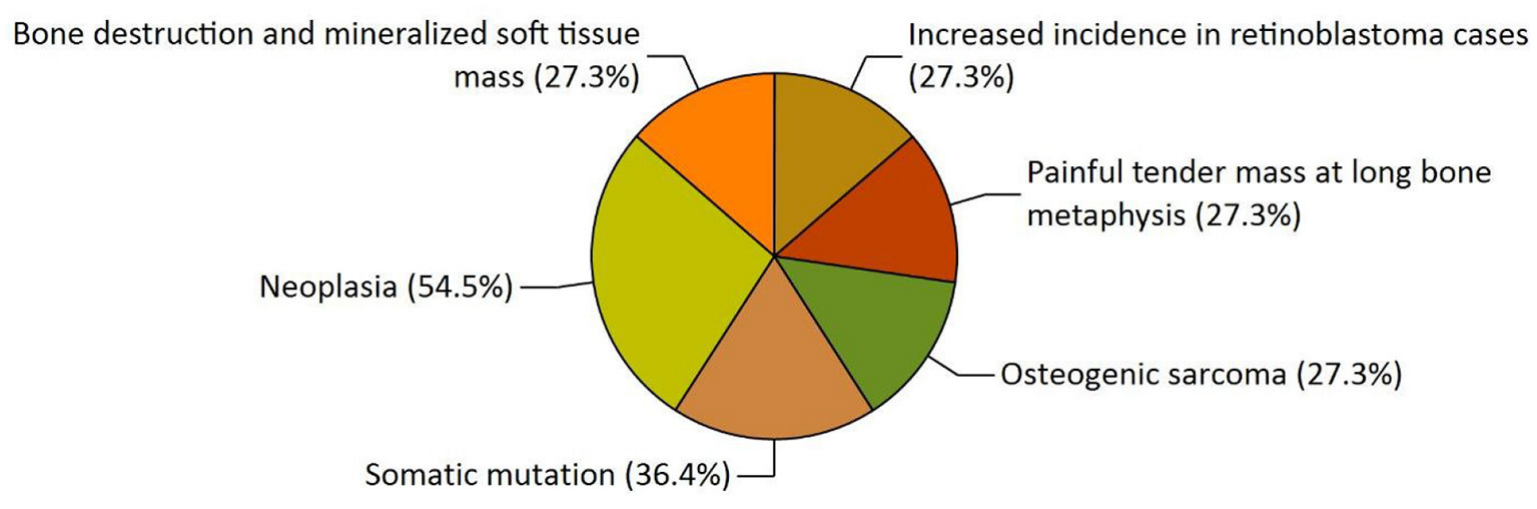
The functional enrichment analysis of osteosarcoma genes performed by the FunRich tool revealed clinical phenotypes.

**Figure 6:**
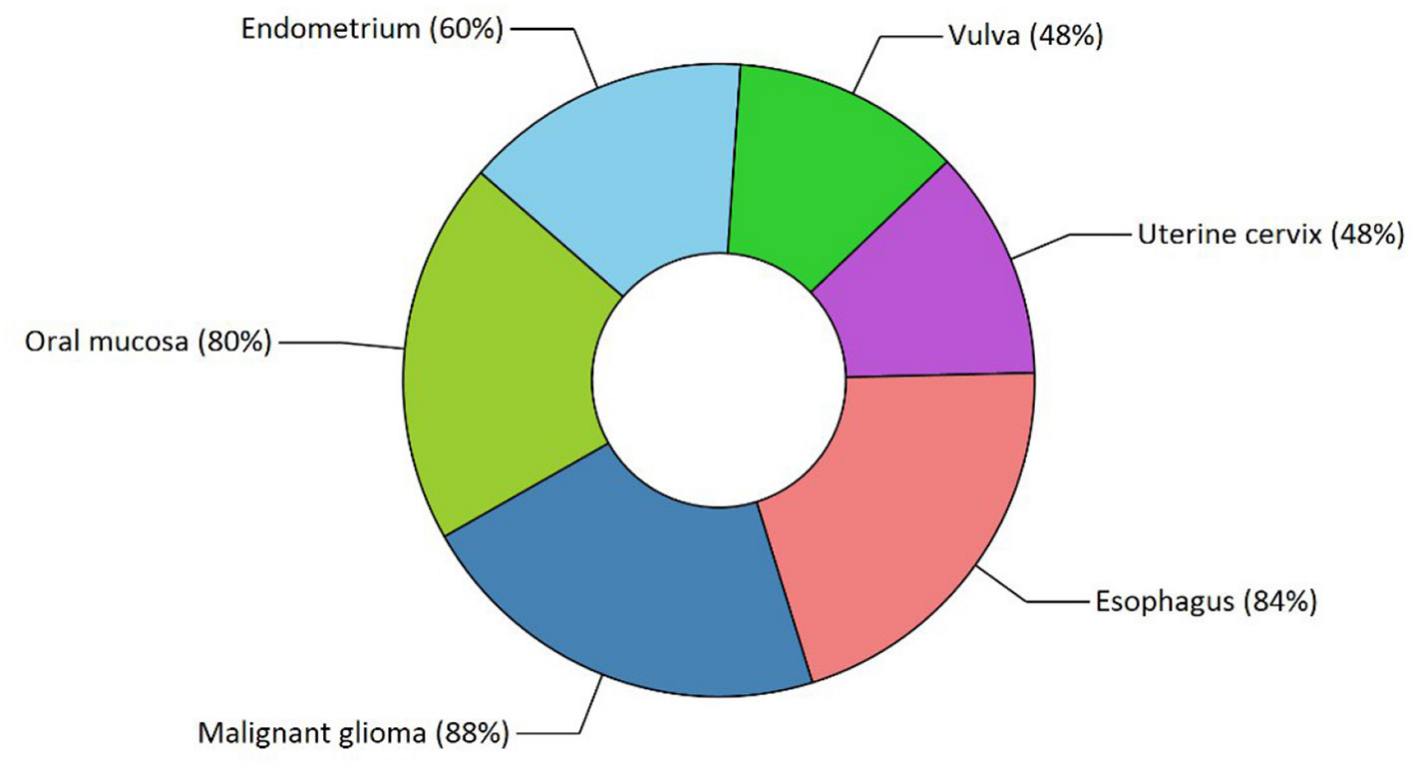
Functional enrichment analysis revealed sites of expression of osteosarcoma genes. The various highlighted portions show different sites of expression.

Our study has also revealed various highly enriched pathways, such as the FOXM1 transcription factor network, ATM pathway, signaling event mediated by FAK, Arf6 signaling events, and Class 1 PI3K signaling events. FOXM1, a Forkhead Box Transcription Factor, is known for maintaining the homoeostatic environment and other cellular functions, such as cell proliferation, cell cycle progression, DNA damage repair, angiogenesis, etc. Being associated with a large number of cellular processes, it has also manifested its role in several diseases as well as cancer. It has been studied that FOXM1 plays a role in tumor growth and progression (52). Forkhead box (Fox) proteins belong to a superfamily of evolutionarily conserved transcriptional factors characterized by a common DNA binding domain known as the forkhead box or winged helix domain (53). FOXM1 preferentially binds promoter regions with the consensus “TAAACA” recognition sequence (54). Cell cycle regulation regulates its expression at mRNA and protein levels. It increases during the S-phase, peaks G2 and M, and degrades during mitotic exit (55). Genetic alteration and gene copy amplification of FOXM1 has been seen at loci 12p13.33, exhibiting oncogenic properties (56). Various studies have highlighted that the alterations arise in FOXM1 during post-transcriptional and post-translational modifications, which leads to its deregulation and overexpression in cancer cells (55, 57). The role of FOXM1 in tumor cells is its participation in the self–renewal and proliferation of cancer stem cells through Wnt signaling, the MAPK-ERK pathway, and the PI3K-mTOR pathway (58). Studies conducted on patients suffering from Osteosarcoma have revealed that the upregulation of miR-370 suppressed the expression of FOXMI. On the contrary, it was also evident that miR-370 was reduced in Osteosarcoma cells where FOXM1 expression was elevated. The miR-370 is a class of micro-RNA involved in various cellular processes such as proliferation, differentiation, apoptosis, and tumor suppression (59). Thus, micro-RNA can serve as a potential drug target in controlling the spread of Osteosarcoma by FOXM1 factor since the contribution of this transcription factor in promoting the disease is exemplary. The Ataxia-Telangiectasia Mutated (ATM) kinase is an essential sensor and signal transducer in the DNA damage response. It is noteworthy that ATM is often considered a major tumor suppressor because of its ability to induce cell cycle arrest. However, certain tumor cells in the advanced stages exhibit enhanced ATM signaling, which benefits cancer cell survival, resistance to radiation and chemotherapy, biosynthesis, proliferation, and metastasis (60). ATM is an active serine/threonine kinase and is an important member of the P13K-related protein kinase family (PIKK). The two main types of ATM signaling are the canonical route, which is activated by DNA damage and signals with the Mre11-Rad50-NBS1 (MRN) complex, and many non-canonical modes of activation triggered by other types of cellular stress. Both types of signaling are likely to play a part in ATM's ability to limit tumor growth (61). PI3K family members such as ATM are routinely auto-inhibited when they are in their resting state (dimers or polymers), and are only activated when they attach to their partners. The ATM canonical pathway is activated upon DNA double-strand breaks (DSBs), where ATM dimers are dissociated to monomers, activation is triggered, and ATM monomers are recruited to the DNA damage sites (62, 63). Since ATMs induce cell cycle arrest and apoptosis whenever genetic alteration occurs, cancer cells use various mechanisms to downregulate ATMs. For instance, ATM expression can be decreased in some cancers due to miRNA-18a (64). Arf6 is a member of the adenosine diphosphate (ADP)-ribosylation factor (ARF) family of small GTPases. By regulating the transit of proteins and lipids in eukaryotic cells, ARFs influence cellular behavior and function (65). Arf6 controls cytoskeletal remodeling, cell shape alterations, extracellular matrix proteolysis, and cell adhesion mechanisms involved in tumor cell migration (66). Degradation of the ECM by matrix metalloproteinases (MMPs) is required for tumor cell invasion. MMPs are released into the extracellular environment by both invadopodia and tumor cell-expelled microvesicles, aiding the breakdown of the ECM and invasion (67). Initiation of Arf6 leads to the activation of Rho and Rac1 pathways, which promotes both microvesicle shedding and formation of invadopodia, whereas expression of a dominant negative Arf6 prevents the development of invadopodia and microvesicle shedding (68, 69). The phosphoinositide 3-kinase (PI3K) family is crucial to almost every aspect of cell and tissue biology and hyperactivation of PI3K is one of the central events in cancer (70). Studies carried out in the early 2000s were the first to show that class I PI3K catalytic isoforms had the ability to alter themselves. Since the discovery of its mutated form, PI3KCA, PI3K has been placed on the frontline as a big player in understanding cancer. The enrichment analysis of Osteosarcoma genes has also revealed clinical phenotypes and sites of gene expression where the former consisted of neoplasia, somatic mutation, osteogenic sarcoma, and painful tender mass at long bone metaphysis (Figure 5) while the latter comprised of the esophagus, oral mucosa, malignant glioma, endometrium, uterine cervix, and vulva (Figure 6).

To identify possible drug targets for Osteosarcoma, which plays an essential role in various biological pathways, we used NetworkAnalyzer, which is a built plugin in Cytoscape. NetworkAnalyzer uses various parameters such as degree, average shortest path length, closeness centrality, and betweenness centrality. Degree refers to the no. of direct interactors, and more no. of degrees will indicate more no. of gene interactors which will help us to understand the progression of a pathway. The significance of the gene in gene-to-gene communication increases with decreasing average shortest path length and increasing closeness centrality. Based on the parameters mentioned above, our study has revealed the top five genes viz. *TP53, CCND1, CDK4, STAT3*, and *VEGFA*, can be considered potential biomarkers because they are involved in the major biological pathways of Osteosarcoma.

*TP53* gene is a potential biomarker with the most no. of direct interactors of 45 with the shortest average path length of 1.343 and the highest closeness centrality of 0.744. *TP53* is seen to be involved in various biological pathways. In our study, such as the FOXM1 transcription factor network, ATM pathway, Class 1 PI3K signaling events, and mTOR signaling pathway. In normal conditions, *TP53* is a tumor suppressor gene that initiates numerous stress-induced pathways, including DNA damage, senescence, cellular death, and reprogramming. It stimulates numerous genes encoding proteins responsible for apoptosis (71). In a cancerous state, *TP53* is mutated, which loses its ability to suppress the tumor, thereby promoting uncontrolled cell proliferation. Over 50% of human neoplasms have somatic mutations in the *TP53* gene. About 10% of the changes are nonsense mutations, resulting in shortened p53 proteins, while most variants are missense mutations. Sixty percent of neoplasms with missense *TP53* mutations have their second *TP53* allele deleted (72). Earlier studies demonstrated that FOXM1 expression is increased when p53 is partially deleted or inactivated by negatively regulating the expression of FOXM1. Similar studies on *TP53* have revealed that reverse regulation of *TP53* through the mTOR pathway also modifies the synchronization of growth signals and stressors (73). The *TP53* gene is considered a hallmark in cancer studies and serves maximum potential for developing therapeutic targets for treating Osteosarcoma.

*CCND1* gene can serve as a drug target with 33 direct interactors having a path length of 1.582 and closeness centrality of 0.632. *CCND1* or *Cyclin D1* gene synthesizes a protein that governs cyclin-dependent kinases in the cell cycle. It is well recognized as a regulator of cell cycle progression in the nucleus, modifying the transition from the G1 to the S phase. Although Cyclin D1 is well recognized for its function in the nucleus, current clinical investigations link it to tumor invasion and metastasis when it is present in the cytoplasmic membrane (74). It is altered in 4.10% of all cancers, typically by post-transcriptional regulation, translocation, or amplification (75).

Additionally, emerging evidence reveals that *CCND1* gene mutations that cause nuclear retention and constitutive CDK4/6 kinase activation are oncogenic (76). *CCND1* is also seen to be involved in biological pathways such as the FOMX1 transcription pathway, mTOR signaling pathway, etc. *CCND1* is seen to be involved in response to leptin, which is a peptide hormone produced by adipocytes. Leptin helps in the maintenance of normal cellular homeostasis. Downregulation of the apoptotic reaction and upregulation of the cell cycle is due to the pro-carcinogenic impact of leptin (77). Therefore, targeting the *CCND1* gene may aid in halting Osteosarcoma development.

*CDK4* gene plays a significant role in the completion of the cell cycle and are often hyperactive in cancer. CDKs are serine/threonine kinases that are activated in association with a cyclin partner. It has a no. of direct interactors of 28 with an average shortest path length of 1.626 and closeness centrality of 0.614. During the G1-S transition, retinoblastoma protein acts as a negative cell cycle regulator by binding to the transcription factor E2F and suppressing transcriptional activity during the early G1 phase. D-type cyclins express themselves more often in response to mitogenic stimuli, and they join forces with CDK4/6 to phosphorylate RB. The E2F transcription factor family's inhibitory control on RB is partially relieved by hypo phosphorylated RB, which encourages the expression of E2F target genes like cyclin E and speeds up the G1 phase transition (78). Studies have also shown that *CDK4* is involved in the regulation of the mTOR pathway activated, thus making it a potential drug target (79).

The *VEGFA* gene is considered a hallmark in cancer-related studies because of its role in angiogenesis, accomplished periodically from pre-existing vascular networks (80). The tumor angiogenesis is achieved in four steps. First is disruption of the basement membrane leading to hypoxia. Second is the dispersion of endothelial cells activated by VEGFA, followed by the proliferation and stabilization of endothelial cells. At last, the angiogenesis regulating factors regulates the repeated process of angiogenesis (81). Studies have also demonstrated that *the* FOXM1 transcription factor regulates *VEGFA* to promote tumor angiogenesis (82). *VEGFA* gene had a degree value of 27 and an average shortest path length of 1.701.

The signal transducer and activator of transcription, *STAT3*, plays a vital role in DNA replication. Being an essential STAT protein family member, it plays a crucial part in various vital cellular functions, including proliferation, differentiation, survival, immunosuppression, angiogenesis, and cancer (83). *STAT3*-activated genes enhance angiogenesis and metastasis, prevent apoptosis, promote cell proliferation and survival, and suppress antitumor immune responses (84). In addition to its established role as a transcription factor in cancer, *STAT3* regulates mitochondrion functions (85). *STAT* gene has been revealed to have direct interactors of 27 with an average shortest path length of 1.716 and closeness centrality of 0.587.

## 5. Conclusion

Osteosarcoma is one of the most frequently occurring sarcomas with a high potency of tumorigenesis. Although chemotherapy and radiotherapy are available as treatment options that have improved patients' lives, there is still some gray area regarding the etiology of the disease. To develop better therapeutics, a specific approach toward the disease targeting the hub genes involved in various signaling pathways must be opted to unravel the complexity of the disease. Our study has mentioned hub genes viz. *TP53, CCND1 CDK4, STAT3*, and *VEGFA* have the highest no. of interactions and showed a high clustering density. Their involvement in the major signaling pathways of Osteosarcoma makes them potential candidates to be targeted for drug development. The highly enriched signaling pathways include the FOXM1 transcription pathway, ATM signaling pathway, FAK mediated signaling events, Arf6 signaling events, Class 1 PI3K signaling events, mTOR signaling pathway, and Integrin family cell surface interactions. Targeting the hub genes and their associated functional partners, which we have reported in our studies, may be efficacious in developing novel therapeutic targets.

## Data availability statement

The original contributions presented in the study are included in the article/Supplementary material, further inquiries can be directed to the corresponding authors.

## Author contributions

Conceptualization: HD, KV, GD, and AE. Methodology: HD and KV. Formal analysis: HD, SK, and AA. Investigation: HD, KV, GD, and HZ. Data curation: HD and SK. Writing—original draft preparation: HD, KV, and AE. Writing—review and editing: KV, AE, GD, and HZ. Supervision: KV and AE. All authors have read and agreed to the published version of the manuscript.
